# Genome-wide identification and characterization of *bHLH* family genes from *Ginkgo biloba*

**DOI:** 10.1038/s41598-020-69305-3

**Published:** 2020-08-13

**Authors:** Xian Zhou, Yongling Liao, Soo-Un Kim, Zexiong Chen, Gongping Nie, Shuiyuan Cheng, Jiabao Ye, Feng Xu

**Affiliations:** 1 College of Horticulture and Gardening, Yangtze University, Jingzhou, 434025 China; 2 Department of Agricultural Biotechnology and Research Institute for Agricultural Sciences, Seoul National University, Seoul, 08826 Republic of Korea; 3 Research Institute for Special Plants, Chongqing University of Arts and Sciences, Chongqing, 402160 China; 4 National R&D for Se-Rich Agricultural Products Processing Technology, Wuhan Polytechnic University, Wuhan, 430023 China; 5 National Selenium Rich Product Quality Supervision and Inspection Center, Enshi, 445000 Hubei China

**Keywords:** Genetics, Molecular biology, Plant sciences

## Abstract

Basic helix–loop–helix (bHLH) proteins, one of the most important and largest transcription factor family in plants, play important roles in regulating growth and development, stress response. In recent years, many bHLH family genes have been identified and characterized in woody plants. However, a systematic analysis of the *bHLH* gene family has not been reported in *Ginkgo biloba*, the oldest relic plant species. In this study, we identifed a total of 85 *GbbHLH* genes from the genomic and transcriptomic databases *of G. biloba*, which were classified into 17 subfamilies based on the phylogenetic analysis. Gene structures analysis indicated that the number of exon–intron range in *GbbHLHs* from 0 to 12. The MEME analysis showed that two conserved motifs, motif 1 and motif 2, distributed in most GbbHLH protein. Subcellular localization analysis exhibited that most GbbHLHs located in nucleus and a few GbbHLHs were distributed in chloroplast, plasma membrane and peroxisome. Promoter *cis-element* analysis revealed that most of the *GbbHLH* genes contained abundant *cis-elements* that involved in plant growth and development, secondary metabolism biosynthesis, various abiotic stresses response. In addition, correlation analysis between gene expression and flavonoid content screened seven candidate *GbbHLH* genes involved in flavonoid biosynthesis, providing the targeted gene encoding transcript factor for increase the flavonoid production through genetic engineering in *G. biloba*.

## Introduction

The basic helix–loop–helix (bHLH) proteins are one of the most important and largest transcription factor families in plants. All the bHLH proteins contain a highly conserved bHLH domain comprised of HLH region and basic region. HLH region is characterized by two α-helices connected by a loop (HLH)^1^. Hence the name is derived from this structural motif. In addition, two α-helices constitutes dimerization motif with approximately 45 amino acids that is indispensable in the formation of bHLH homodimers or heterodimers^2,3^. Generally, the basic region with approximately 15 amino acids facilitates binding to DNA^2^. At present, a large number of *bHLH* gene family have been identified and characterized at genome-wide level from some plant species, such as *Arabidopsis thaliana*^4^, *Phyllostachys edulis*^5^, *Daucus carota*^6^, and *Panax ginseng*^7^.

The bHLH classifications have been improved continuously as the functions of bHLH proteins were determined. bHLH are typically classified into six major groups from A to F according to sequence similarity and evolutionary relationship and the ability to bind DNA^8,9^. Group A mainly binds to the E-box (CAGCTG or CACCTG), which acts as neural and mesodermal development^10^. Group B binds to G-box (CACGTG), which is involved in the expression of glucose-responsive genes and the sterol metabolism^11^. Group C contain bHLH domain and PAS domain that bind ACGTG or GCGTG sequences, which are involved in developmental signaling and environmental homeostasis^12^. Group D lacks a basic region and binds to group A formatting heterodimers^11^. Group E bind to N boxes (CACGCG or CACGAG) that function as embryonic segmentation, somitogenesis and organogenesis^13^. Group F contains COE domain except bHLH domain for dimerization and DNA binding, which is related to head development and formation of olfactory sensory neurons^11^.

*Ginkgo biloba*, one of relic plant species, is looked as one living fossil^14^, contains flavonoids and terpenoids that affect antioxidant activities, platelet-activating factors, peripheral blood vessels, and blood circulation^15^. Flavonoids are synthesized by the combination of the phenylpropanoid and polyketide pathways^16^.Transcription factor were involved in flavonoid biosynthesis by regulating expression of structural genes^17^. Some structural genes related to flavonoid biosynthesis were cloned and characterized from *G. biloba*, including phenylalanine ammonia-lyase (*PAL*)^18^, flavonol synthase (*FLS*)^19^, flavanone 3-hydroxylase (*F3H*)^20^, chalcone synthase (*CHS*)^21^, chalcone isomerase (*CHI*)^22^, isoflavone reductase-like (*IFR-like*)^23^, dihydroflavonol-4-reductase (*DFR*)^24^, anthocyanidin reductase (*ANR*)^25^, anthocyanidin synthase (*ANS*)^26^, cinnamate‑4‑hydroxylase (*C4H*)^27^. In addition, the transcription factors (bHLH, MYB, and WD40) were also reported to play important role in the biosynthetic pathway of flavonoids^28^. Although some literatures reported the genome-wide map and second generation and full-length transcriptome analysis related to related flavonoids biosynthesis in *G. biloba*^29–32^, little information about *bHLH* genes is available in *G. biloba*. In the present study, we used bioinformatics to identify the *bHLH* family gene members and analyzed the relevant characteristics of these family members based on reported genomic sequencing and full-length transcriptome databases. In addition, we screened some *bHLH* genes which might be involved in biosynthetic pathway of flavonoids in *G. biloba*. Our data provided the targeted gene resource of transcript factor involved in flavonoids biosynthesis for increase the flavonoid production through genetic engineering in *G. biloba*.

## Result

### Identification and physicochemical properties of bHLH proteins from *G. biloba*

**Figure 1:**
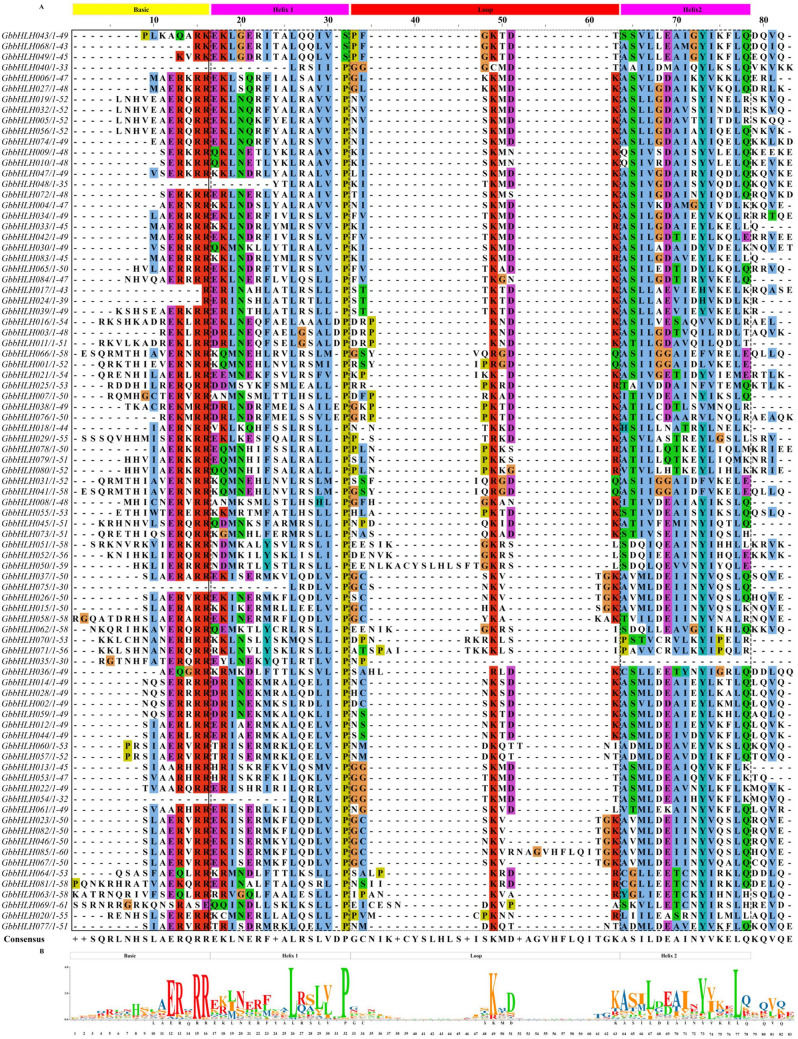
Multiple alignment of conserved domain amino acid sequences of multiple bHLH proteins from *G. biloba*. (**A**) Multiple sequence alignment of convserved bHLH domain of bHLH proteins from *G. biloba*. Alignment was carried out using Clustal W and represented by Adobe ExtendScript Toolkit CS6. (**B**) Analysis of bHLH domain motif by TBtools. Highly conserved amino acid residues in the bHLH domain across all GbbHLHs. The conservation of the sequence at that position was represents height of each stack.

Here, a combined analysis of genome-wide and full-length transcriptome-wide was carried out to screen and identify *bHLH* genes in *G. biloba* using the publicly available genomic sequences and our recently published full-length transcriptome data^32,33^. A total of 85 putative bHLH proteins (GbbHLH) were obtained based on reported genomic sequencing and full-length transcriptome databases of *G. biloba* (Tables S1, S2). To further characterize these GbbHLHs, we analyzed the physicochemical properties of the putative proteins. These 85 GbbHLH proteins showed diversities in length, molecular weight, theoretical isoelectric points (PIs), number of negatively charged residues (Asp and Glu), and number of positively charged residues (Arg and Lys) (Table S2). Specifically, the lengths of the 85 GbbHLH proteins ranged from 98 to 1,469 amino acid residues, while their pIs were between 4.74 and 9.39 with an average of 6.78 (Table S2). The grand average of hydropathicity of the candidate GbbHLH proteins ranged from − 0.856 to 0.514. Most of GbbHLH proteins belonged to hydrophilic characteristics, except for GbbHLH042. The multiple sequence alignment of bHLH domain sequence of GbbHLH proteins showed that the basic region and two helixes were highly conserved in most of GbbHLH proteins, except the basic region was absent in GbbHLH040, GbbHLH048, GbbHLH054 and GbbHLH075, and the helix 2 region was absent in GbbHLH035 (Fig. 1A). Among amino acids of conserved bHLH domain, nineteen amino acid residues were highly conserved (> 50% consensus ratio), and eight of those were conserved with a > 75% consensus ratio. Moreover, basic region (Glu-12, Arg-13, Arg-15and Arg-16) consensus ratio were higher than 75%, helix 1 region (Leu-26, Leu-29, Val-30 and Pro-31), loop region(Asp-50 and Lys-51) and helix 2 region (Ala-52, Ser-53, Leu-55,Glu-57, Ala-58, Ile-59, Tyr-61 and Leu-65) consensus ratio beyond to 50% (Fig. 1B).

### Evolutionary tree analysis of *bHLH* gene family

**Figure 2:**
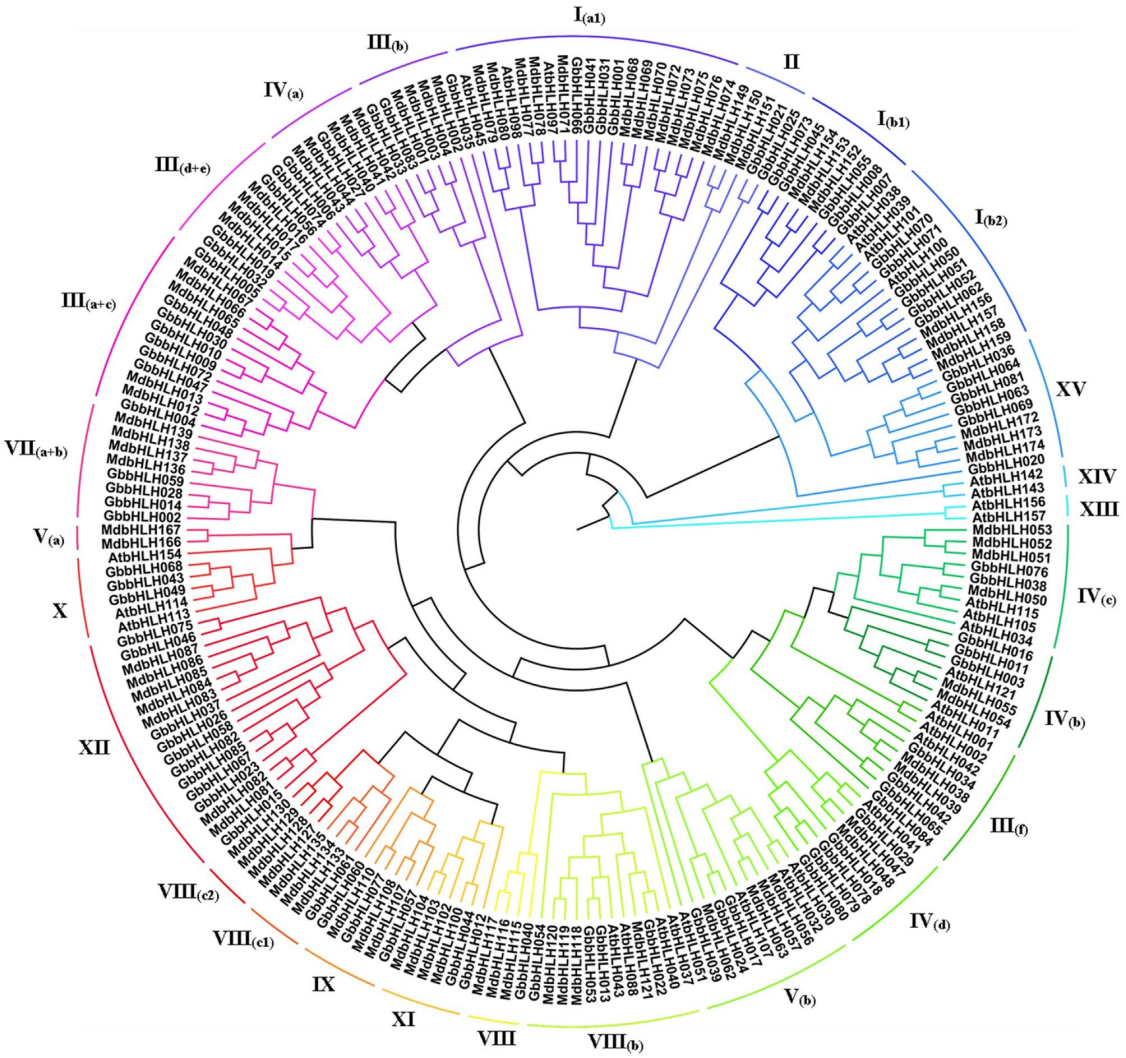
Phylogenetic tree constructed using the sequences of bHLH domain proteins from *Arabidopsis thaliana*, *Manus domestica* and *G. biloba*. The tree was generated using Clustal X2 and MEGA 6 by using neighbor-joining method with 1,000 bootstrap replicates. All *bHLH* genes are clustered into subclades based on the priority classification rule of Arabidopsis *bHLH* genes.

To classify the *G. biloba* bHLH protein subfamilies and identify the evolutionary relationships among the bHLH proteins from *G. biloba*, *Manus domestica*, and *A. thaliana*, a phylogenetic tree were constructed using the sequences of the 85 GbbHLH proteins, 94 MdbHLH proteins, and 11 *A. thaliana* bHLH proteins. As shown in Fig. 2, the 85 bHLH members of *G. biloba* clustered into 17 subfamilies according to the topology of the tree and classification of the bHLH superfamily in *A. thaliana* and *M. domestica*. The 17 subfamilies were designated as I(a1), I(b1), I(b2), II, III(a + c), III b, III (d + e), IIIf, IVa, IVb, IVc, IVd, Vb, VII(a + b), VIII, VIIIb, VIII(c1), IX, X, XI, XII, and XV (Fig. 2). None of the *G. biloba* bHLH proteins were grouped into subfamilies V(a), VI, VIII(c2), XIII, and XIV possibly due to the loss of these proteins during the evolution of *G. biloba*. In sum, the number of *G. biloba* bHLHs within each subfamily varied from 1 to 10.

### Gene structure and characterization of conserved bHLH motifs from *G. biloba*

**Figure 3:**
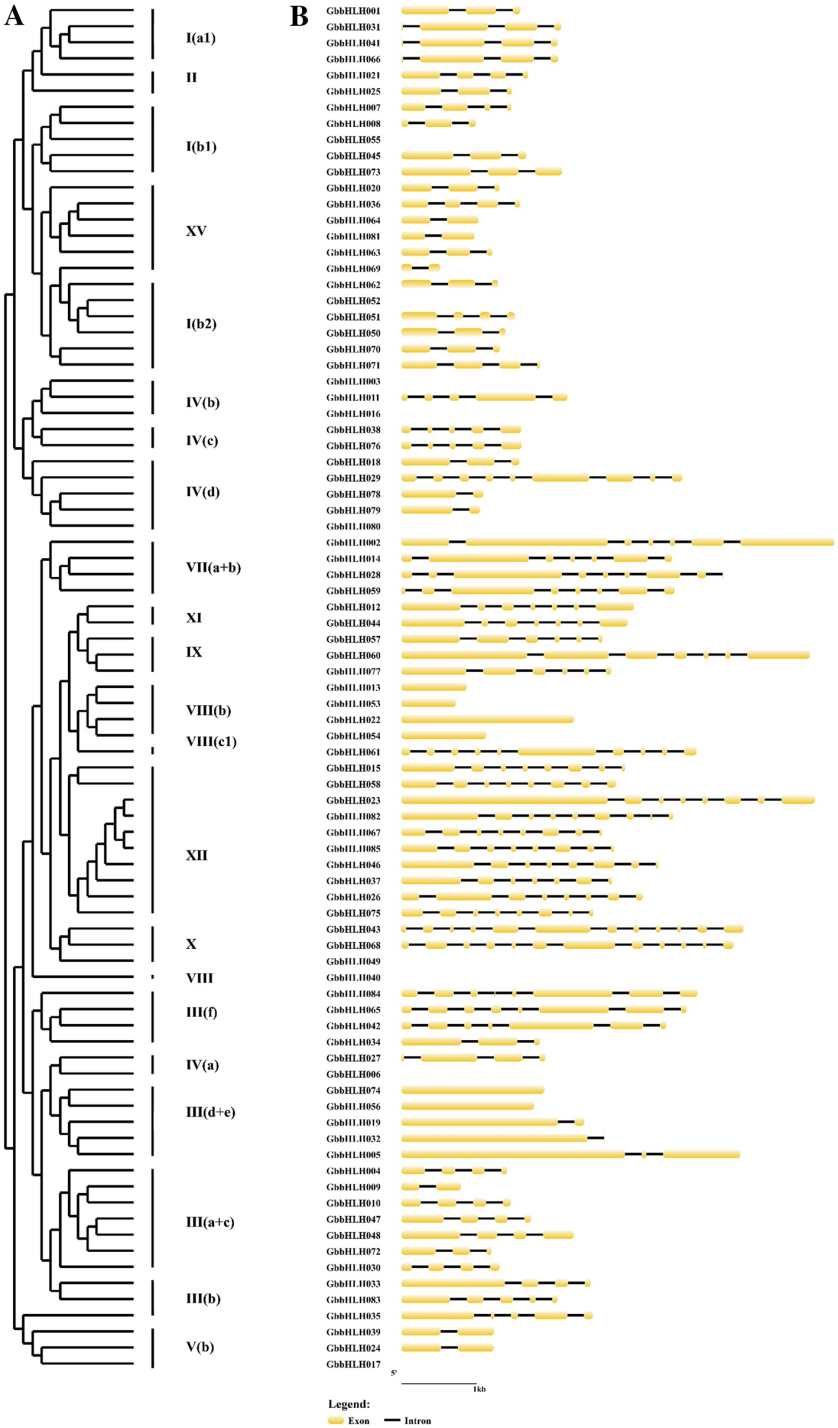
Phylogenetic relationship and gene structure analysis of *bHLH* genes in *G. biloba*. (**A**) phylogenetic tree was constructed from the alignment of amino acid sequencing of selected bHLH proteins from *G. biloba*. (**B**) Gene structure analysis of selected *bHLH* genes of *G. biloba*, showing locations and lengths of the exons and introns. Exons and introns are presented as filled yellow round-corner rectangle and thin single lines, respectively.

The schematic gene structures of *GbbHLH* genes were analyzed using the GSDS tool (Fig. 3). Among 85 *GbbHLHs*, 75 were identified from the genomic database. Therefore, we analyzed the exon–intron distribution of 75 *GbbHLHs* of *G. biloba*. The 75 *GbbHLH* genes had a varying number of exons from 1 to 12. Among these *GbbHLHs*, 6 gene members, that is *GbbHLH013, GbbHLH022, GbbHLH 053, GbbHLH054, GbbHLH056*, and *GbbHLH074*, were intron-less and distributed across VIII(b) and III(d + e). Five gene members, *GbbHLH003, GbbHLH011, GbbHLH038*, and *GbbHLH076* of subfamilies IV(b) and IV(c), were predicted to exhibit five exons and four introns, respectively. Two members (*GbbHLH044* and *GbbHLH012* from subfamily XI) exhibited seven exons and six introns, respectively. The members of subfamily V(b) exhibited two exons and one intron. The members of subfamilies III(a + c), III(b), I(a1), I(b1), I(b2), and XV presented two to five exons and one to four introns. The members of subfamilies III(f), VII(a + b), and XII presented six to nine exons and five to eight introns. *GbbHLH043* and *GbbHLH068* exhibited 12 exons and 11 introns.

**Figure 4:**
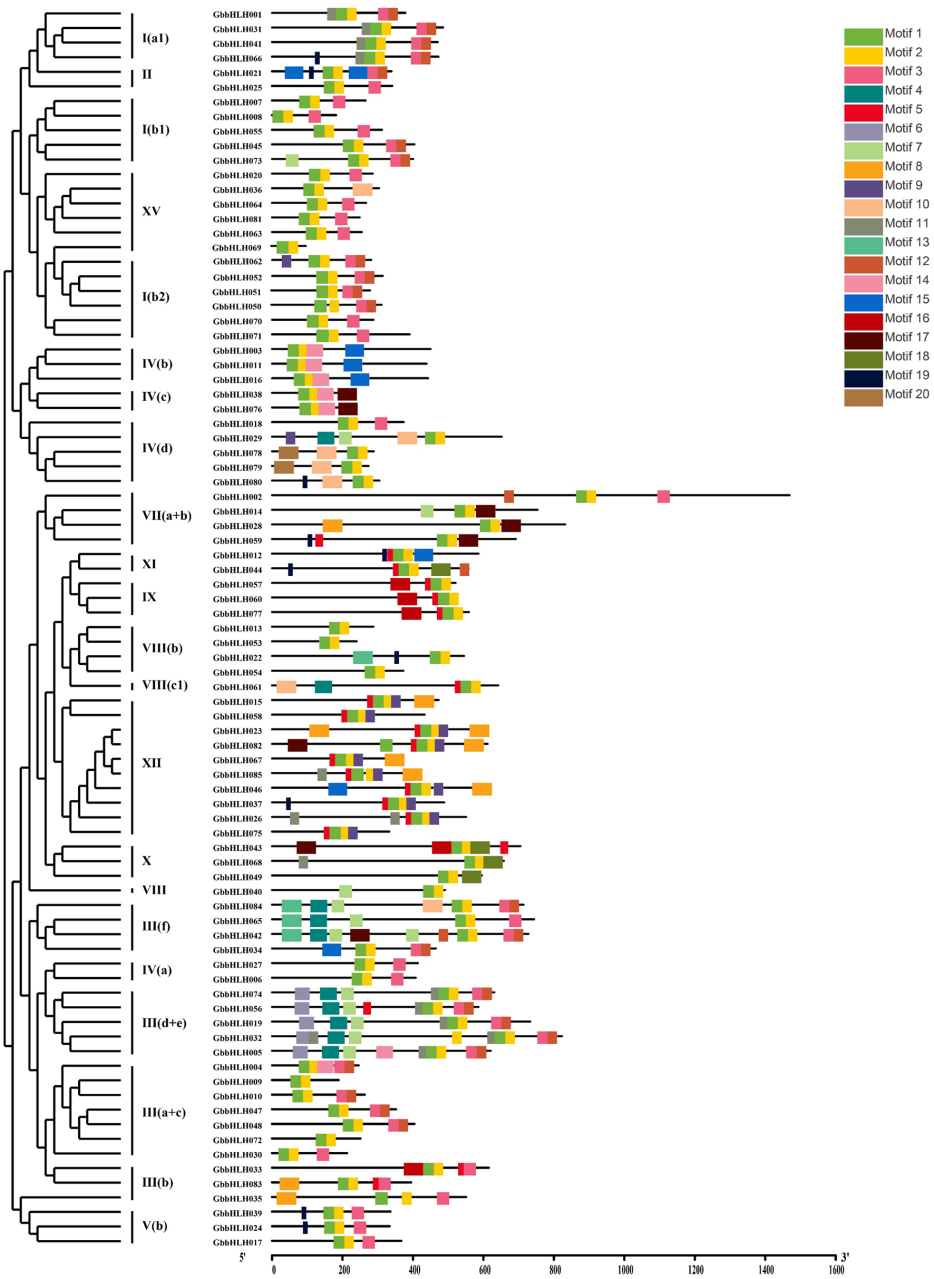
Motif composition and distribution of 85 bHLH proteins in *G. biloba*. The motifs of the GbbHLH proteins were analyzed using the MEME web server. The length of the black line indicates the length of a sequence relative to all the other sequences. The position of each block indicates the location of a motif with a matching sequence.

MEME analysis showed that all GbbHLH proteins except GbbHLH060 contained highly conserved Gb-motif 1 and Gb-motif 2, which consists of 15 and 29 amino acids, respectively (Figs. 4 and S1). The Gb-motifs belonging to the same subfamily of bHLHs were the same or similar. GbbHLH038 and GbbHLH076 from the subfamily IV(c) contained 4 motifs, while GbbHLH003, GbbHLH011, and GbbHLH016 from subfamilies I(b2) and IV(b) all contained four motifs. Most Gb-motifs, such as subfamilies I(b2), III(a + c), IV(a), IV(c), V(b), VII(a + b), VIII(b), and XV, were located near the C-terminus. However, Some Gb-motifs, such as those found in subfamily IV(b), were located near the N-terminus.

### Analysis of GO annotation and subcellular localization

**Figure 5:**
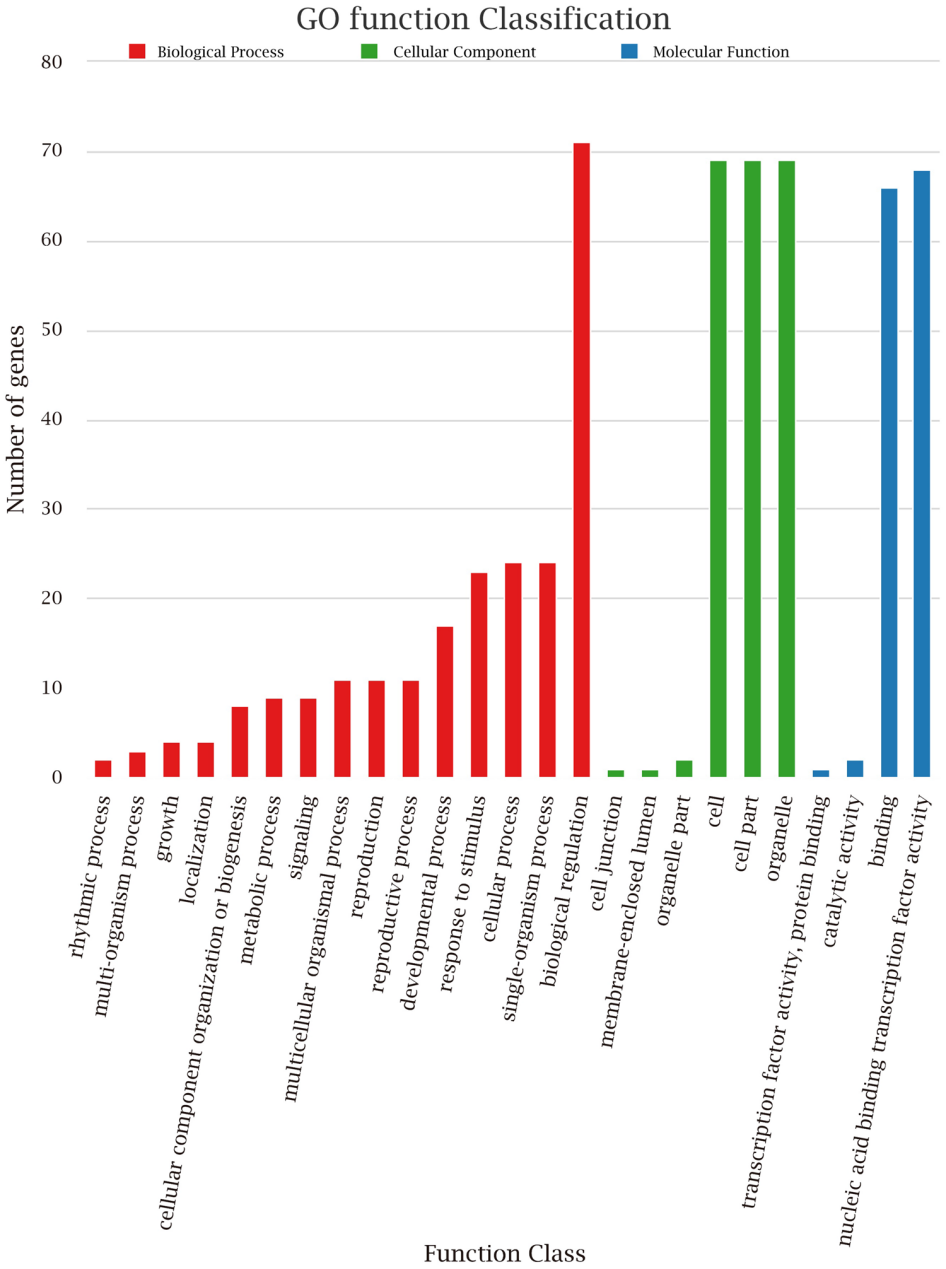
The Go annotation of *bHLH* genes in *G. biloba*. All annotated GO terms including biological process, cellular component and molecular function of 85 GbbHLHs.

The GO annotation of *GbbHLHs* showed three aspects of functional classifications, namely, molecular function, cellular component, and biological process (Fig. 5). *GbbHLH002*, *GbbHLH024*, and *GbbHLH069* were annotated in the molecular function, which is related to transcriptional regulation. Only *GbbHLH069* was annotated in the cellular component. Among 85 bHLH members, 14 *GbbHLH* genes, including *GbbHLH002*, *GbbHLH009*, *GbbHLH023*, *GbbHLH024*, *GbbHLH032*, *GbbHLH038*, *GbbHLH039*, *GbbHLH043*, *GbbHLH056*, *GbbHLH060*, GbbHLH069, *GbbHLH072*, *GbbHLH073*, and *GbbHLH076*, were annotated in biological process and involved in DNA binding, oxidoreductase activity, and protein dimerization activity.

The remaining 71 *GbbHLH* genes cannot be annotated to GO databases, which accounts for 83.53% of the total *GbbHLH* genes. We conducted annotated 71 *GbbHLH* genes according to evolution of *G. biloba*, *Manus domestica*, and *A. thaliana.* We found that the number of bHLH genes involved in biological regulation was up to 71. Among these genes, 69 *bHLH* genes were involved in cell, cell part and organelle, respectively. A total of 66 *bHLH* genes and 68 *bHLH* genes were classified into binding nucleic acid and binding transcription factor activity, respectively.

The subcellular localization analysis of the 85 bHLH protein were performed online with WOLF PSORT. As shown in Table S3, a total of 74 GbbHLHs were predicted to located in the nucleus (up to 87%), 8 of which, including GbbHLH015, GbbHLH020, GbbHLH051, GbbHLH061, GbbHLH062, GbbHLH063, GbbHLH064, and GbbHLH068, were predicted to located in the chloroplast (0.09%). Only GbbHLH035 was supposed to located in the plasma membrane. GbbHLH022 and GbbHLH083 were likely to located in the peroxisome.

### Promoter analysis and protein–protein interaction network prediction

**Figure 6:**
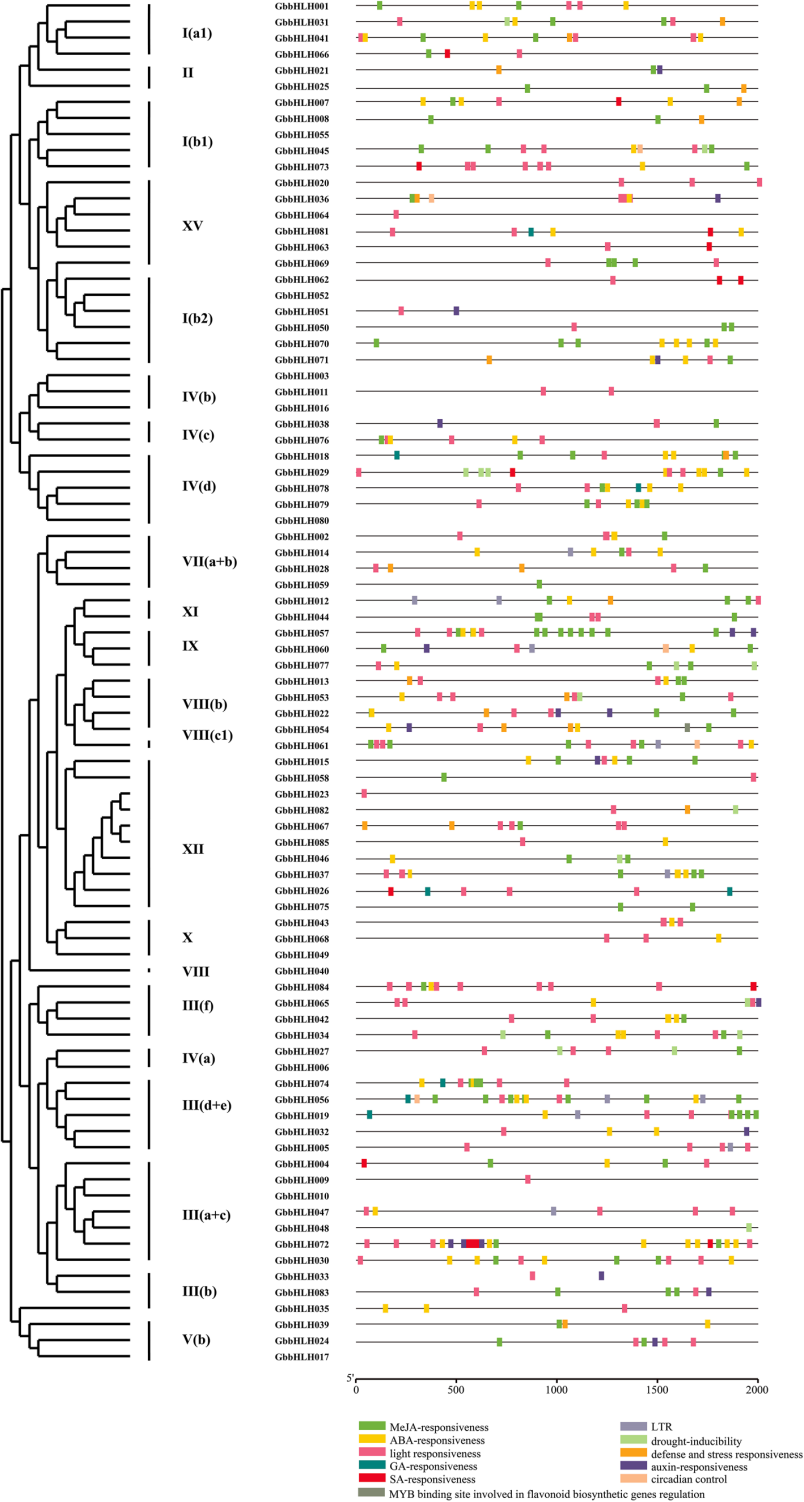
*Cis*-element analysis of 74 *bHLH* gene promoters in *G. biloba*. The potential cis-regulatory elements in the promoter regions 2,000 bp upstream of the *G. biloba* were predicted by PlantCARE software. Different colors indicated the elements related to growth and development (circadian control), plant hormones (abscisic acid, auxin, methyl jasmonate, gibberellic acid, and salicylic acid) and stress responsiveness (anaerobic induction, light, low temperature, and drought inducibility).

Many *bHLH* genes play important roles in plant growth and development, as well as in response to various abiotic stresses. To further investigate the putative functions of *GbbHLH* genes, we identified and analyzed the potential *cis-elements* in the promoter regions of 2000-bp upstream of the start codon of bHLH genes using PlantCARE software. As shown in Fig. 6, three main categories were found in the *cis-elements* of *GbbHLH* genes. Category one was related to plant growth and development, such as cell differentiation, circadian control, and cell cycle regulation. This category was composed of ARE, AT-rich sequence, HD-Zip-1, RY-element, GCN4_motif, AACA_motif, circadian, and MSA-like. Category two was involved in phytohormones, such as abscisic acid (ABA), auxin, gibberellin, methyl jasmonate (MeJA), and salicylic acid (SA). This category included ABA response element (ABRE), AuxRR-core, CGTCA-motif, TATC-box, TCA-element, and TGACG-motif. Category three was associated with abiotic stresses, such as light responsiveness, drought inducibility, wound responsiveness, anaerobic induction, and low-temperature responsiveness. Category three contained 3-AF1 binding site, AAAC-motif, ACE, C-box, G-Box, GT1-motif, LTR, MBS, MRE, P-box, Sp1, TC-rich repeats, and WUN-motif.

**Figure 7:**
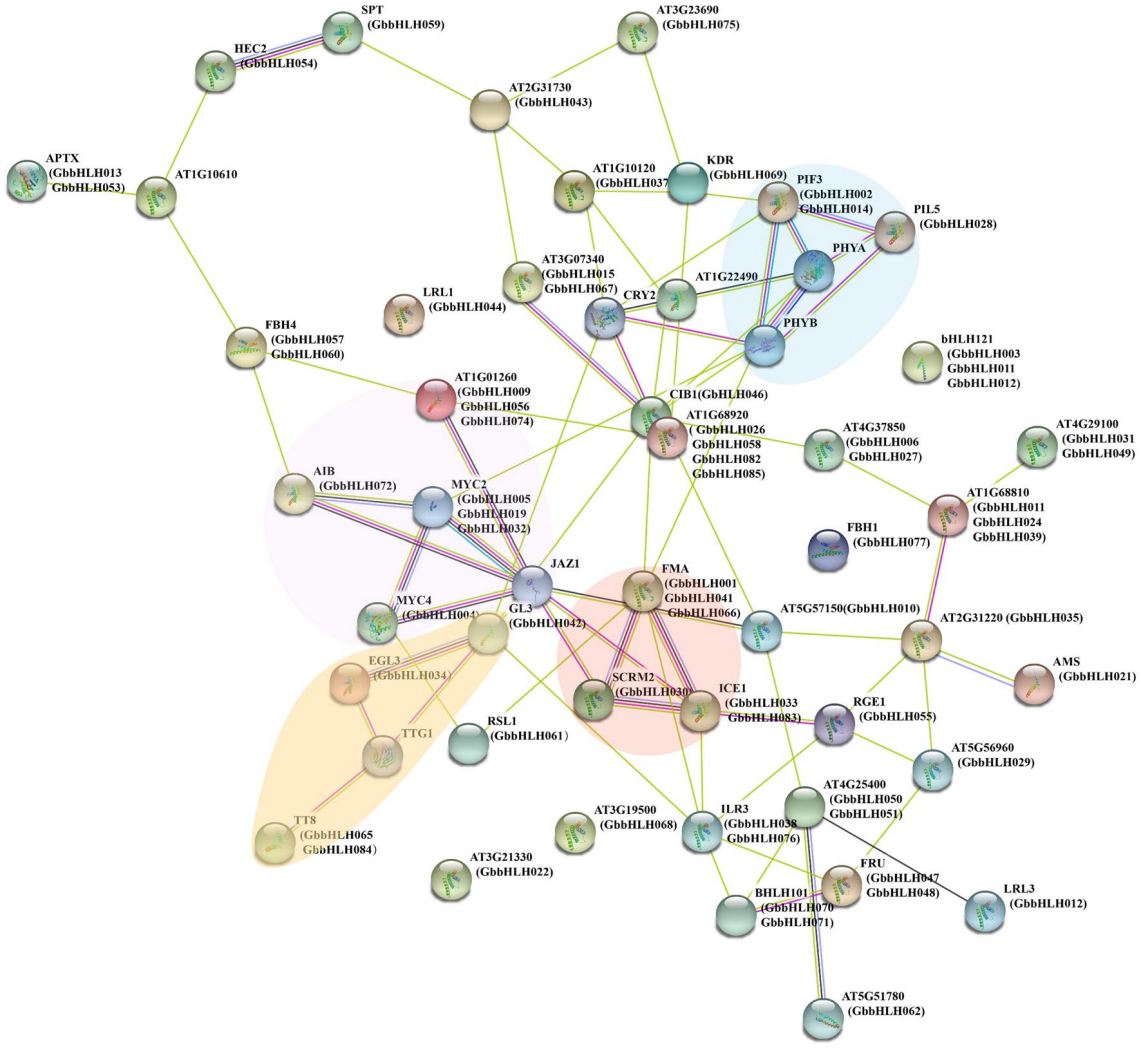
Functional regulatory network of 85 *G. biloba* bHLH proteins. The protein–protein interaction of bHLH proteins was predicted using STRING software. Cyan line presents data from curated databases, purple line experimentally determined, green line gene neighborhood, red line gene fusions, blue line gene co-occurrence; yellow line presents text mining, black line co-expression and gray line protein homology.

The interaction of 85 GbbHLHs was predicted by STRING (Fig. 7). In the protein sequence homology to *A. thaliana*, GbbHLH002 and GbbHLH014 that were homologous with PHYTOCHROME-INTERACTING FACTOR 3 (PIF3) belonged to the subfamily VII(a + b) group. GbbHLH028 [homologous PIF3-LIKE 5 (PIL5)] belonged to subfamily VII(a + b) group. PIF3 and PIL5 are related to light signaling and phytohormones signals^33^. GbbHLH028 could interact with GbbHLH002 and GbbHLH014 that regulated light signaling and phytohormones signals pathway GbbHLH005, GbbHLH019, and GbbHLH032 (homologous MYC2) belonged to subfamily III(d + e) group; and GbbHLH004 (homologous MYC4) belonged to subfamily III(a + c) group. MYC2, MYC3 and MYC4 controls additively jasmonate-related defense responses by reducing expression of GS biosynthesis genes. MYC interact directly with GS-related MYBs to regulation of defense secondary metabolite production^34^. Hence, we speculate GbbHLH004 could interact with GbbHLH005, GbbHLH019, and GbbHLH032. That involved in jasmonate-related defense responses. GbbHLH001, GbbHLH041 and GbbHLH066 were homologous with FMA. GbbHLH033and GbbHLH083 were homologous with ICE1. GbbHLH030 was homologous with SCRM2. GbbHLH001, GbbHLH041 and GbbHLH066 could interact with GbbHLH033and GbbHLH083. GbbHLH030 could interact with GbbHLH033and GbbHLH083.

### Candidate bHLHs involved in flavonoids biosynthesis in *G. biloba*

**Figure 8:**
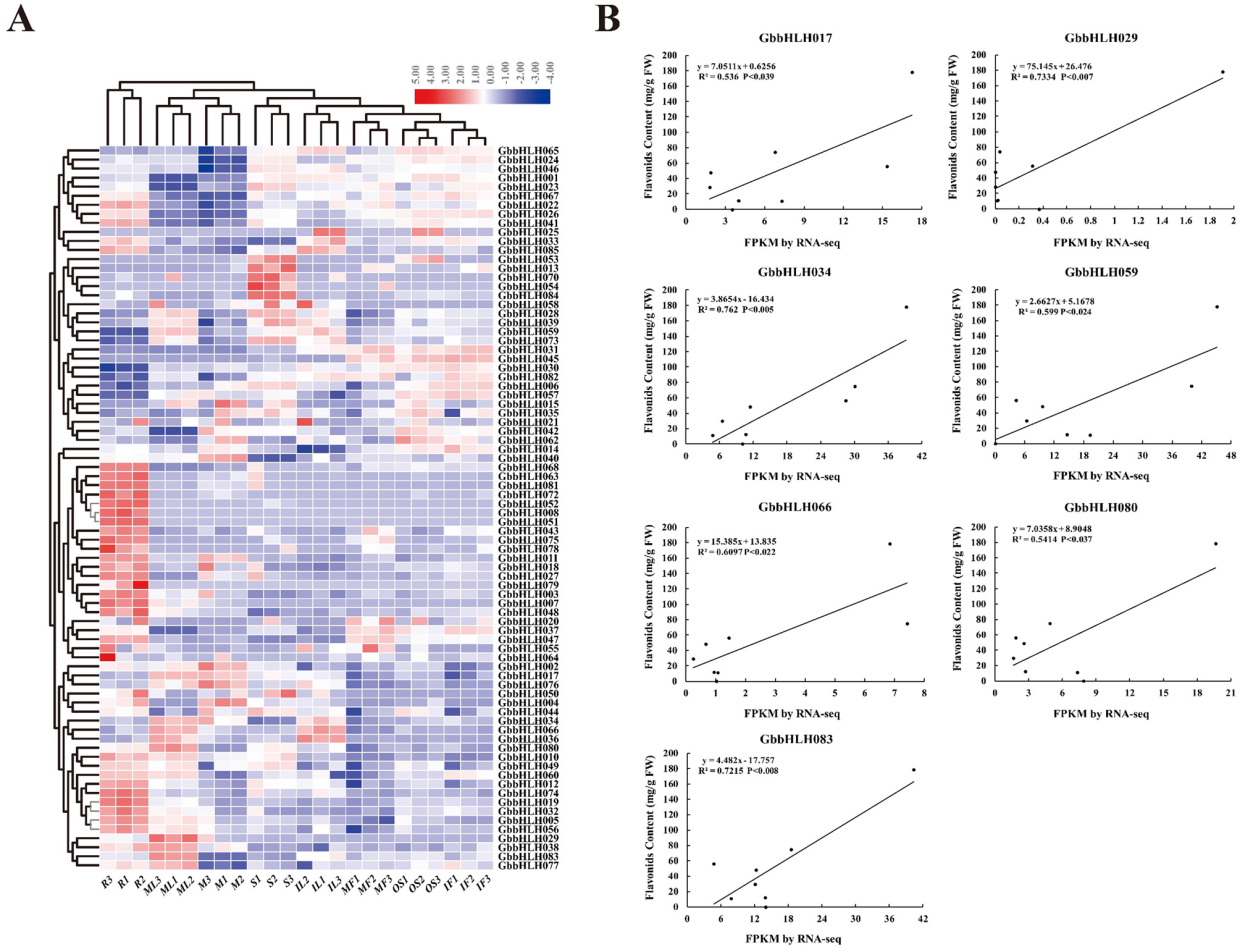
Expression of *GbbHLH* genes and correlation analysis between expression level of *GbbHLH* genes and the content of flavonoids in different organs of *G. biloba*. (**A**) A heatmap shows expression level of 80 *GbbHLH* genes with different subfamilies (left column) in different organs (bottom row) of *G. biloba*. Expression differences are observed in different colors. The R, S, IL, ML, M, OS, IF and MF represent root, stem, immature leaf, mature leaf, microstrobilus, ovulate strobilus, immature fruit and mature fruit, respectively. Changes in expression level are indicated by a change in color; green indicates a lower expression level, whereas red indicates a higher expression level. All data shown reflect the average mean of three biological replicates (n = 3). (**B**): Correlation analysis between the expression level of selected 7 *GbbHLH* genes and the content of flavonoids and in different organs of *G. biloba*.

Our previous work performed Illuminate sequencing of 24 independent cDNA libraries of eight organs (root, stem, immature leaf, mature leaf, microstrobilus, ovulate strobilus, immature fruit and mature fruit) from *G. biloba* with three biological replicates each organ^31^. Based on the RNA-seq data, a total of 80 *GbbHLHs* were expressed in eight different organs of *G. biloba* (Fig. 8A). No expression was observed in *GbbHLH009*, *GbbHLH016*, *GbbHLH061*, *GbbHLH069* and *GbbHLH071.* The spatial expression patterns of 80 *GbbHLHs* were diverse. *GbbHLH041, GbbHLH047, GbbHLH056, GbbHLH068* and *GbbHLH081* were predominantly expressed in root. *GbbHLH040* and *GbbHLH076* were preferentially expressed in microstrobilus. *GbbHLH084* was highly expressed in stem. *GbbHLH083* was mainly expressed in mature leaves. *GbbHLH045* was highly expressed in immature fruit. Based on correlation analysis between the expression level of *GbbHLHs* and flavonoids content using OmicShare tools, the flavonoids content was significantly correlated with expression levels of seven *GbbHLHs* in eight organs of *G. biloba*. In detail, the expression levels of *GbbHLH034* (R^2^ = 0.536), *GbbHLH029* (R^2^ = 0.733), *GbbHLH083* (R^2^ = 0.762), *GbbHLH066* (R^2^ = 0.599), *GbbHLH059* (R^2^ = 0.610), *GbbHLH080* (R^2^ = 0.541) and *GbbHLH017* (R^2^ = 0.722) had significant positive correlation with flavonoids content (*p* < 0.05) (Fig. 8B). Therefore, we suggested that these 7 *bHLH* genes might be involved in flavonoids biosynthesis in *G. biloba*.

#### Chromosomal distribution of *GbbHLH* genes

**Figure 9:**
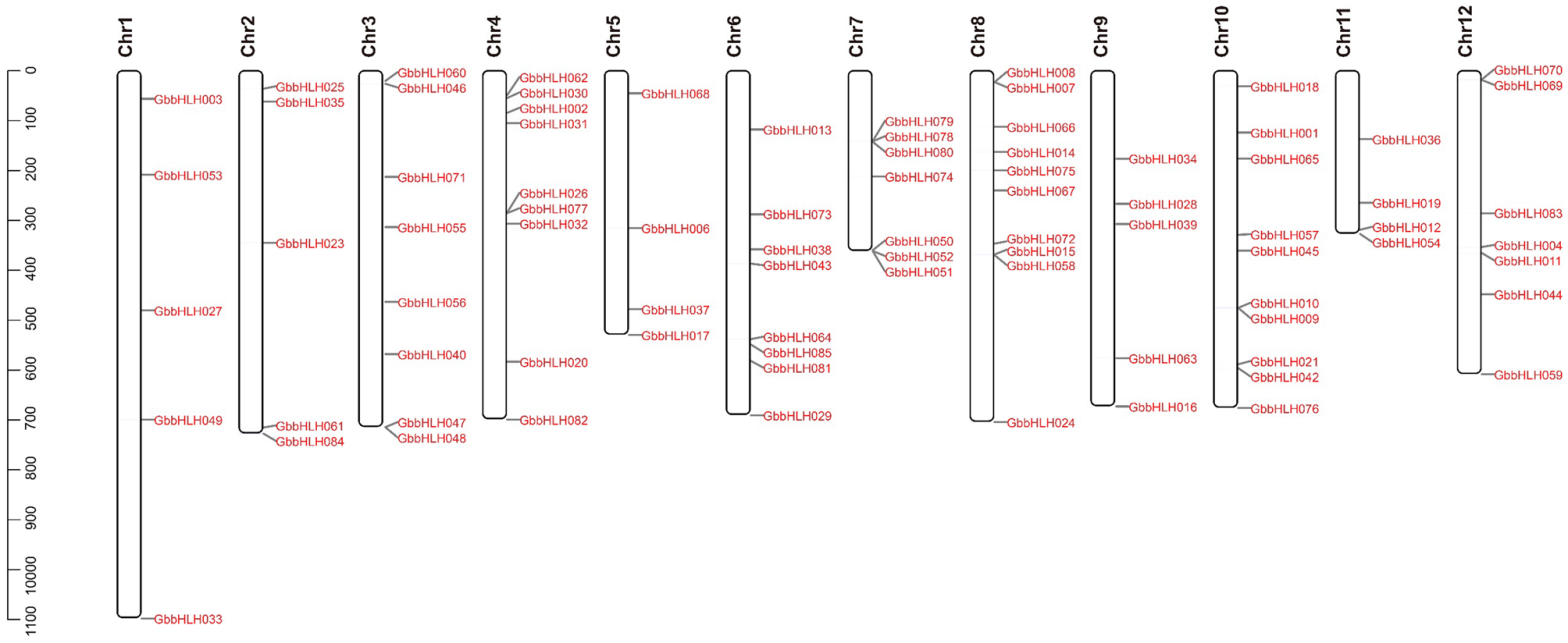
Chromosomal distribution and regional duplication of 82 *bHLH* genes of *G. biloba*. The scale bar on the left indicated the length (Mb) of ginkgo chromosomes.

To characterize the chromosomal distribution of these GbbHLH genes, we integrated 12 scaffolds of the *G. biloba* genome (named Chr.1 to Chr. 12) from the genome database^32^. Among these *GbbHLH* genes, 82 members were successfully mapped to the ginkgo chromosomes (Fig. 9). The number of *bHLH* genes range from 4 to10 in chromosome 1 to 12. Chromosome 8 and chromosome 10 contain 10 *bHLH* genes. Chromosome 5 and chromosome 11 contain 4 *bHLH* genes. In particular, *GbbHLH022* was mapped onto the hic_scaffold_9926 of ginkgo and GbbHLH041 was mapped onto the hic_scaffold_22302 of ginkgo.

## Discussion

The bHLH family genes were previously divided into 21 subfamilies in *A. thaliana*^4^, 23 in *M. domestica*^35^, and 19 in peach^36^. The evolutionary analysis identified 85 *bHLH* genes in *G. biloba*, which were divided into 17 subfamilies. Our results on GbbHLHs showed similarities as well as differences compared to the classifications of the other plant species^35^. In general, the structures and functions of GbbHLH matched with those of other species. In other words, genes with the same or similar functions were clustered on the same branch. For example, AtbHLH045, AtbHLH097, and AtbHLH098 from I(a) subfamily are related to stomatal development control^35,36^. GbbHLH001, GbbHLH031, GbbHLH041, and GbbHLH066 were classified under the subfamily I(a). Thus, these four bHLH genes of *G. biloba* were deduced to participated in stomatal development control. AtbHLH037, AtbHLH040, AtbHLH043, and AtbHLH088 from subfamily VIII(b) regulate flower and fruit development^37^. Likewise, GbbHLH013, GbbHLH022, GbbHLH053 and GbbHLH054, being classified into VIII(b) subfamily, were projected to exhibit similar functions. Previous studies showed that AtbHLH038, AtbHLH039, AtbHLH100 and AtbHLH101 genes are involved in Fe-deficiency response^38^. Hence, the functions of GbbHLH050, GbbHLH051, GbbHLH052, GbbHLH070, and GbbHLH071 from I(b2) subfamily may be analogous as function in Fe-deficiency response of *G. biloba*. Same analogy existed in anthocyanin-related AtbHLH001, AtbHLH002, and AtbHLH042 from subfamily III(f)^39^ and GbbHLH034, GbbHLH042, GbbHLH065and GbbHLH084. Taken together, the evolutionary analysis results and *bHLH* genes with known functions can be combined to predict the *GbbHLH* genes related to growth and development, secondary metabolism, and environmental responses in *G. biloba*.

Gene structures analysis provides important information on phylogenetic relationships. The numbers of exon/intron of the same subfamilies are the same or similar^40^. The exon/intron diversification of gene family members play an important role in the evolution of multiple gene families through the three main types of mechanisms, namely exon/intron gain/loss, exonization/pseudoexonization, and insertion/deletion^41^. The number of exons/introns ranges from 0 to 4 in rice^42^ and from 0 to 19 in apple^35^. In this study, the number of exons/introns in *bHLH* family member of *G. biloba* ranged from 0 to 12, indicating that the exons/introns of *bHLH* genes underwent loss or insertion during the evolution of *G. biloba*. In addition to Exon–intron structures, motif structure also expounds on phylogenetic relationships. The *bHLH* genes of one cluster contained the same or similar motifs as in *P. edulis*^5^. Similar to these results, our study also revealed that motifs 1 and 2 were located in all *GbbHLH* proteins. Therefore, motifs 1 and 2 are important characteristics for identifying ginkgo *bHLH* gene.

Subcellular localization can help to understand location of protein function. Cheng et al.^5^ performed bHLH protein prediction that most bHLH proteins are located in the nucleus, and some bHLH proteins are located in the mitochondria and cytoplasm in *P. edulis*. Similarly, GbbHLH proteins were mainly located in the nucleus. The minor number of the GbbHLH proteins were distributed in the chloroplast, plasma membrane, and peroxisome. These results indicated that GbbHLH proteins might play role in nucleus of *G. biloba*. A small difference in the location of bHLH proteins was also observed between *P. edulis* and *G. biloba*.

Plant promoters are important regulatory elements required for plant gene transcription and play important regulatory roles at the transcriptional level^43^. ABA response elements (ABRE) include ACGTG (*A. thaliana*), GACACGTGGC (*Triticum aestivum*), and CGTACGTGCA (*Hordeum vulgare*), are involved in ABA responsiveness^44^. Moreover, SA responsiveness (TCA element), light responsiveness cis-element (ACE and MRE), circadian control, LTR cis-element of low-temperature responsiveness, and GT-1 box were also reported^45,46^. Secondary metabolites synthesis is subjected to phytohormone regulation (MeJA, SA, ABA, and Eth) and low temperature stresses^47^. Consistent with above reports, our results demonstrated that the promoters of *GbbHLHs* contained numerous and various *cis-elements* that are likely related to plant growth and development regulation and response to various abiotic stresses. In general, bHLH proteins interact with other proteins to function in plant development and metabolism. For example, An et al.^48^ found that MdTTG1 interacts with bHLH proteins to regulate anthocyanin accumulation, while *bHLH* gene *ICE1* interacts with *SPCH*, *FAMA*, and *MUTE* to regulate stomatal cell differentiation in *Arabidopsis*^49^. In addition, phytochrome-interacting factor (*PIF*), proteinase-activated receptor (*PAR*) and paclobutrazol resistance (*PRE*) were members of *bHLH* gene family. *PAR1-PRE1* and *PAR1-PIF4* form heterodimers regulated cell elongation and plant development in response to light and hormones^50^. In our study, some protein–protein interaction among bHLH members was also predicted to be associated with growth and development, abiotic stress, phytohormone, and secondary metabolite.

Flavonoids biosynthesis pathway has been extensively studied^51,52^. In *G. biloba*, several transcription factors were cloned and identified to be involved in biosynthetic pathway of flavonoids. For example, our previous work demonstrated that an *R2R3-MYB* gene *GbMYBF2* act as negative regulators of flavonoids biosynthesis in *G. biloba*^53^. More recently, Zhang et al.^54^ stated that *another MYB* gene *GbMYBFL* played a positive role on flavonoids biosynthesis in *G. biloba*. To date, some bHLH proteins was found to play important role in the regulation of flavonoids biosynthesis. For instance, *bHLH* genes were involved in flavonoids biosynthesis in *Nicotiana tabacum*^55^ and *Chrysanthemum morifolium* Rama^56^. The *VvMYC1* gene encoding bHLH transcription factor interacts with MYB to regulate the expression of three flavonoids biosynthetic genes, including *ANR*, *UFGT*, and *CHI*^57^. bHLH, WD40, and MYB proteins also regulate flavonoids biosynthesis by forming complexes^45,50^. In this study, our data revealed that 7 *GbbHLH* genes were significantly correlated with flavonoids content, implying *GbbHLH* genes that might be involved in flavonoids biosynthesis in *G. biloba*. However, since this conclusion was based on the correlation analysis between the expression levels and flavonoids content, additional experimental information is necessary to establish the claim. The further study could include transgenic research and transcription factor interaction with promoters of key structural genes related to biosynthetic pathway in *G. biloba*.

## Materials and methods

### Identification and classification of *bHLH* genes in *G. biloba*

A local protein database of *G. biloba* was created by the obtained genomic sequences and transcriptome sequences, genomic database comes from *Ginkgo biloba* GigaScience Database. (https://doi.org/10.5524/100.209)^32^, transcriptome sequences database was obtained from NCBI (https://www.ncbi.nlm.nih.gov/sra). The accession is no. SRR7948405 ~ SRR7948413 and SRP149113^31^. The bHLH proteins of *A. thaliana*, and *Malus domestica* were downloaded from the PlantTFDB (https://planttfdb.cbi.pku.edu.cn/prediction.php). The bHLH proteins were blasted by matching the 2 species (*E* value of 0.01) by Bioedit software^58^, and the bHLH proteins were searched against HMMER3.1 software (https://megasoftware.net/) by the hidden Markov model file of the HLH domain (PF00010) that was downloaded from Pfam database (https://pfam.xfam.org)^59^. The bHLH proteins that contain multiple termination signals and repeats were removed. Then, the rest of the bHLH protein were checked in the websites SMART (https://smart.embl-heidelberg.de/) and CDD-Search (https://www.ncbi.nlm.nih.gov/Structure/cdd/wrpsb.cgi) and showed that they remained present in the conserved bHLH domain^60,61^. All bHLH protein sequences were analyzed through bioinformatics analyses, including the prediction of ORFs and physico-chemical properties such as MW, pI, total number of negatively charged residues (Asp + Glu), and total number of positively charged residues (Arg + Lys) using ExPASy (https://web.expasy.org/protparam/)^62^.

### Phylogenetic analysis

Phylogenetic tree was constructed by Clustal X2 and MEGA 6 using neighbor-joining method with bootstrap test (1,000 replicates), Poisson model, and partial deletion^63,64^.

### Gene structure analysis and conserved motif characterization

The exon–intron structures of *GbbHLH* genes was displayed by GSDS (https://gsds.cbi.pku.edu.cn/index.php)^65^. The conserved motifs of the bHLH proteins were searched in MEME 5.0.5 (https://meme.sdsc.edu/meme/) with a maximum of 20 motifs and analyzed by TB tools^66,67^.

### Gene ontology (GO) annotation and subcellular localization prediction

The translated bHLH protein sequences from the full-length transcriptome of *G. biloba*^31^ were annotated using the Blast2GO program to assign the GO terms (https://amigo.geneontology.org/amigo/term/)^68^. The GO analysis showed that the E-value was 1.0E-6, and GO terms were provided under three main categories, namely, biological process, cellular component, and molecular function. The bHLH proteins were uploaded to WOLF PSORT^69^ (https://www.genscript.com/psort.html) to predict subcellular localization.

### Promoter analysis and protein–protein interaction network prediction

The upstream 2,000 bp genomic DNA sequences of the *bHLH* gene start code were downloaded and submitted to PlantCARE to predict putative *cis-elements*^68,70^. The protein–protein interaction of bHLH proteins was predicted using STRING (https://string-db.org/) under the following parameters: *A. thaliana* was selected to perform the comparison analysis, and then the minimum required interaction score was set to middle confidence, that is, 0.400^71^.

### Determination of flavonoids in G. biloba

The flavonoid contents in roots, stems, immature leaves, mature leaves, microstrobilus, ovulate strobilus, immature fruits, and mature fruits were determined according to the method of Ye et al.^29^. The flavonoid contents were calculated by multiplying the total content of quercetin, kaempferol, and isorhamnetin with a factor of 2.51, and were expressed as percentage (m/m)^72^.

### Correlation analysis between flavonoid content and gene expression level

Our previous work constructed 24 independent cDNA libraries of eight organs (root, stem, immature leaf, mature leaf, microstrobilus, ovulate strobilus, immature fruit and mature fruit) from *G. biloba* with three biological replicates each organ^31^. The 24 cDNA libraries were sequenced using an Illumina Hiseq X Ten Platform by Biomarker Biotechnology (Beijing, China). The SRA accession of these sequencing raw data is nos. SRR7948405–SRR7948413 in NCBI (https://www.ncbi.nlm.nih.gov/sra). The gene expression levels were estimated by fragments per kilobase of transcript per million fragments mapped with the following equation: FPKM = cDNA Fragments/[Mapped Fragments (Millions) × Transcript Length (kb)]. Flavonoids content and expression levels of *GbbHLH* genes were performed to correlation analysis by applying OmicShare tools (https://www.omicshare.com/tools) to identify genes involved in flavonoids metabolism with correlation coeffcients of ≥ 0.6. Thus, r > 0.6 and P < 0.05 meant significant correlation were considered to have an expression that was significantly correlated with the expression of genes in the biosynthetic pathways of flavonoids.

### The location of bHLH genes on chromosomes

The position of each *GbbHLH* gene on the twelve chromosomes was obtained from the GigaDB site (https://gigadb.org/dataset/100613) and was visualized using TBtools^67^.

## Conclusion

In this study, we identified 85 *GbbHLH*s through HMMER and BLAST from *G. biloba*. These *GbbHLH* genes were classified into 17 subfamilies by comparative phylogenetic analysis with *A. thaliana* and *M. domestica* bHLH proteins. Meanwhile, exon/intron and motif analyses supported the results of phylogenetic analysis. A total 74 GbbHLHs were predicted to locate in the nucleus, while other 11 GbbHLHs were located in the chloroplast, plasma membrane, and peroxisome, respectively. The *cis-elements* in the *G. biloba bHLH* gene promoters were identified to be related to phytohormone and abiotic stresses. The protein–protein interaction prediction results indicated that GbbHLH proteins are involved in phytohormone. Finally, the correlation analysis between gene expression and flavonoid content revealed seven candidate *GbbHLH* genes involved in flavonoids biosynthesis. The results of our study provide a foundation for understanding molecular mechanism of bHLH regulating flavonoids biosynthesis in *G. biloba*.

## Supplementary information

Supplementary Figure S1

Supplementary Table S1

Supplementary Table S2

Supplementary Table S3
