# Supplementary figures and images for: Genome-wide identification and characterization of bHLH family genes from Ginkgo biloba
Source: Sci Rep. 2020 Aug 13;10:13723. doi: 10.1038/s41598-020-69305-3 (PMC7426926; doi:10.1038/s41598-020-69305-3)

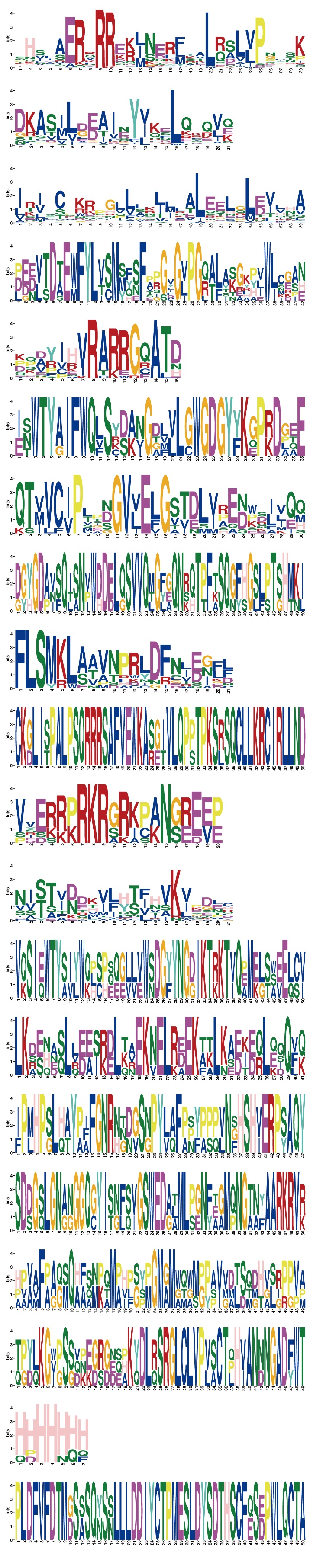


**Figure S1.** Sequence information of 85 bHLHprotein matching each motif in *G. biloba*.

Supplement: Supplementary file 1 — Supplementary Figure S1 [file 41598_2020_69305_MOESM1_ESM.docx]
